# Supplementary material for: Deep Learning Automated Segmentation for Muscle and Adipose Tissue from Abdominal Computed Tomography in Polytrauma Patients
Source: Sensors (Basel). 2021 Mar 16;21(6):2083. doi: 10.3390/s21062083 (PMC8002279; doi:10.3390/s21062083)
Supplement: Supplementary file 1 [file sensors-21-02083-s001.pdf]

Supplementary

Deep Learning Automated Segmentation for Muscle and Adipose Tissue from Abdominal Computed Tomography in Polytrauma Patients

Table S1. Hyper-parameters for deep learning network training.

Hyper-Parameter	Setting
Training-validation partition for model development	Randomly-assigned;<br>80% training (n = 2729)<br>20% validation (n = 684)
Convolution kernels	3 x 3, window 1 (all except final layer)<br>1 x 1, window 1 (final layer)
Number of deep features	Block 1 & 9 : 16<br>Block 2 & 8 : 32<br>Block 3 & 7 : 64<br>Block 4 & 6 : 128<br>Block 5 : 256<br>Final convolution layer : 4
Activation functions	Parametric ReLU (all except final layer)<br>Softmax (final layer)
Optimizer	Adam
Loss function	Categorical cross-entropy
Learning rate	0.0001
Dropout	0.05
L2 (ridge) regularization penalty	0.001
Max-pooling	2 x 2, strides 2
Batch normalization	Momentum 0.99; Epsilon 0.001
Early stopping criteria	Patience 2000 and delta 0.001

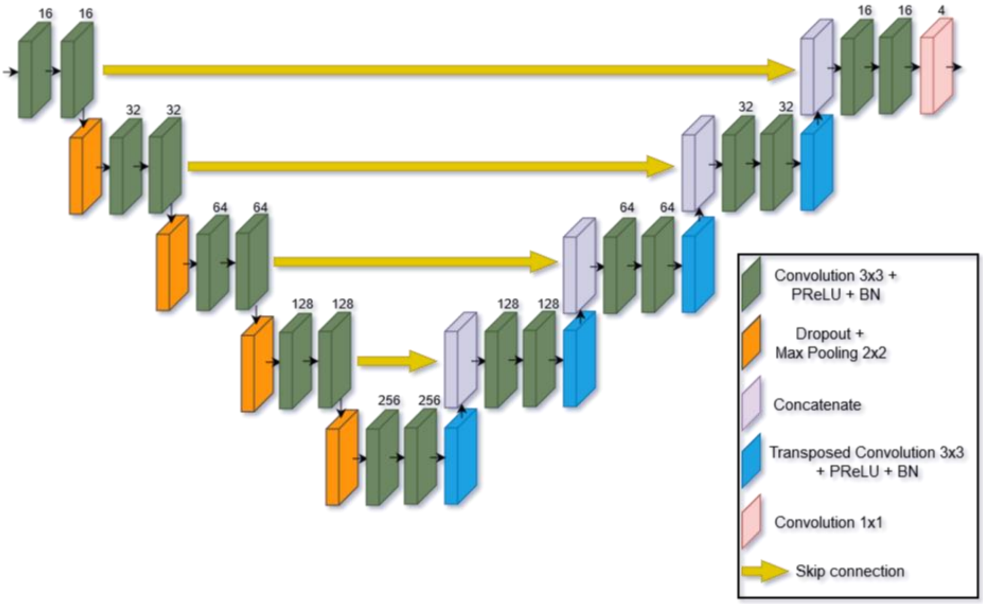

Figure S1. Schematic block diagram showing the architecture of the deep learning neural network for automatic segmentation
